# Absence of cardiac impairment in patients after severe acute respiratory syndrome coronavirus type 2 infection: A long-term follow-up study

**DOI:** 10.1016/j.jocmr.2024.101124

**Published:** 2024-11-15

**Authors:** Janek Salatzki, Andreas Ochs, Lukas D. Weberling, Jannick Heins, Marc Zahlten, James G. Whayne, Christian Stehning, Evangelos Giannitsis, Claudia M. Denkinger, Uta Merle, Sebastian J. Buss, Henning Steen, Florian André, Norbert Frey

**Affiliations:** a Department of Cardiology, Angiology and Pneumology, University Hospital Heidelberg, Heidelberg, Germany; b DZHK (German Centre for Cardiovascular Research), partner site Heidelberg, Heidelberg, Germany; c Myocardial Solutions Inc., Morrisville, North Carolina, USA; d Philips Clinical Science, Hamburg, Germany; e Division of Infectious Disease and Tropical Medicine, University Hospital Heidelberg, Heidelberg, Germany; f German Center of Infection Research, partner site Heidelberg, Heidelberg, Germany; g Department of Gastroenterology, Infectious Diseases and Intoxication, University Hospital Heidelberg, Heidelberg, Germany; h MVZ DRZ Heidelberg, Heidelberg, Germany; i medneo, Hamburg, Germany

**Keywords:** CMR, SARS-CoV-2, COVID, FSENC, Cardiac impairment, Persistent symptom

## Abstract

**Background:**

Concerns exist that long-term cardiac alterations occur after severe acute respiratory syndrome coronavirus type 2 (SARS-CoV-2) infection, particularly in patients who were hospitalized in the acute phase or who remain symptomatic. This study investigates potential long-term functional and morphological alterations after SARS-CoV-2 infection.

**Methods:**

The authors of this study investigated patients after SARS-CoV-2 infection by using a mobile 1.5T clinical magnetic resonance scanner for cardiac alterations. Cardiac function and dimensions were assessed using a highly efficient cardiac magnetic resonance protocol, which included cine sequences, global longitudinal and circumferential strain assessed by fast-Strain-ENCoded imaging, and T1 and T2 mapping. We assessed symptoms through a questionnaire. Patients were compared with a control group matched for age, gender, body mass index, and body surface area.

**Results:**

Median follow-up time was 395 (192-408) days. The final population included 183 participants (age 48.4 ± 14.3 years, 48.1% male (88/183)). During the acute phase of SARS-CoV-2 infection, 27 patients were hospital-admitted. Forty-two patients reported persistent symptoms (shortness of breath, chest pain, palpitations, or leg edema), and 63 reported impaired exercise tolerance. Left ventricular (LV) functional and morphological parameters were within the normal range. T1- and T2-relaxation times were also within the normal range, indicating that the presence of myocardial edema or fibrosis was unlikely. Persistently symptomatic patients showed a slightly reduced indexed LV stroke volume. Functional parameters remained normal in patients who were hospitalized for SARS-CoV-2, persistently symptomatic, or with ongoing impaired exercise tolerance.

**Conclusion:**

Irrespective of ongoing symptoms or severity of prior illness, patients who have recovered from SARS-CoV-2 infection demonstrate normal functional and morphological cardiac parameters. Long-term cardiac changes due to SARS-CoV-2 infection appear to be rare.

## Graphical abstract

**Figure:**
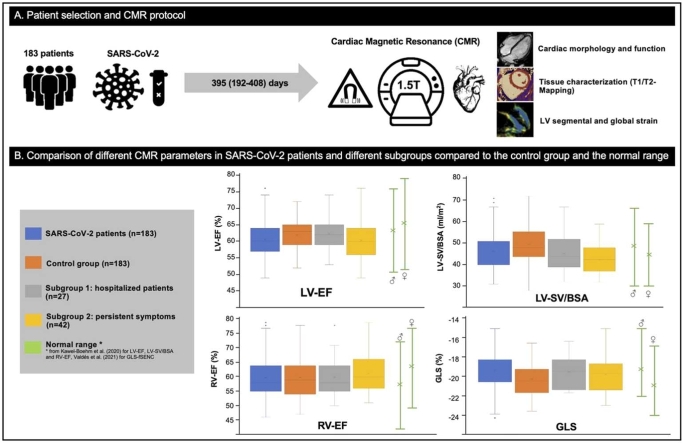

## 1 Background

The coronavirus disease 2019 (COVID-19) pandemic posed unprecedented challenges to health care systems, significantly impacting morbidity and mortality [1]. The long-term cardiac effects of COVID-19, however, are still unclear [1]. Several studies have investigated the cardiac impact of COVID-19, including a wide range of disease severity, from asymptomatic cases to severe acute myocarditis affecting left ventricular (LV) function [2], [3], [4], [5]. Recent data have shown that some patients with even mild symptoms have persistent abnormalities on cardiac magnetic resonance imaging (CMR); nevertheless, the clinical relevance of COVID-19 infection is controversial [6].

Cardiovascular magnetic resonance (CMR) is a powerful imaging tool for the detection of myocardial changes [7], [8]. It is not only the current non-invasive gold standard for the assessment of cardiac morphology and function, but it also allows for tissue characterization to detect myocardial scar, fibrosis, or edema. Experts have therefore recommended CMR for the diagnosis and risk stratification of various cardiac diseases, such as cardiomyopathies or myocarditis [9], [10].

Myocardial strain analysis based on CMR has been shown to improve to the diagnosis and to more precisely predict risk in different cardiac conditions such as heart failure or myocarditis [11], [12]. Fast-Strain-ENCoded imaging (fSENC) accurately measures segmental strain. fSENC is thus able to identify even minor changes in myocardial strain for early identification of heart failure patients, without the use of a contrast agent [13]. Furthermore, T1 and T2 mapping have emerged as contrast agent-free techniques in the differential diagnosis of myocarditis or different forms of cardiomyopathies [14], [15].

This study investigates possible functional and structural cardiac alterations in a large cohort of patients after severe acute respiratory syndrome coronavirus type 2 (SARS-CoV-2) infection using highly efficient, contrast agent-free CMR.

## 2 Methods

### 2.1 Study subjects

Patients who suffered from SARS-CoV-2 infection were prospectively enrolled in this study and examined in April and May 2021. Inclusion criterion included an acute SARS-CoV-2 infection at least 4 months before enrollment, confirmed through either a positive polymerase chain reaction test for SARS-CoV-2 or serologic detection of SARS-CoV-2 antibodies. Exclusion criteria included a known history of heart failure, cardiomyopathy, prior myocarditis, prior myocardial infarction, significant coronary artery disease (CAD) with history of coronary intervention or coronary bypass grafting, or implantation of cardiac devices. Notably, patients with confirmed non-obstructive CAD, as determined by invasive or computed tomography coronary angiography, were not excluded from the study.

All participants underwent an examination, including a detailed medical history, physical examination, and 12-lead electrocardiogram. This study used a questionnaire to gather a detailed history of each participant's SARS-CoV-2 infections, including any symptoms and any hospitalizations that occurred during those infections.

### 2.2 Selection of controls

The control group consisted of individuals without SARS-CoV-2 infection and without known heart disease, and therefore exhibited normal values for LV and right ventricular (RV) function, volumes, and LV strain. Those individuals were obtained from the Department of Cardiology, Angiology and Pneumology of the Heidelberg University Hospital and from the Marien Hospital Hamburg, Germany. Most individuals in the control group were examined before April 2020; only 12 out of 183 were examined in May and June 2020.

The authors matched study subjects to the individuals of the control group according to age, gender, body mass index (BMI), and body surface area (BSA).

Standard CMR was performed with 1.5T or 3T clinical non-mobile scanners (Ingenia CX, Achieva or Ingenia, Philips Healthcare, Best, The Netherlands). A separate control group of 11 individuals without SARS-CoV-2 infection and free of cardiovascular disease was used to compare native T1 times from the 1.5T mobile scanner with the diseased cohort. Functional parameters were measured in all control group patients. Native T1 and T2 times were additionally compared with the existing academic literature [16], [17].

### 2.3 Ethics agreements

All SARS-CoV-2 patients gave written informed consent, and the study was approved by the local institutional ethics committee (S-270/2021). For patients in the control group, informed consent was waived by the local institutional ethics committee (S-101/2019). All procedures complied with the Declaration of Helsinki.

### 2.4 Image acquisition and analysis

All CMR examinations of SARS-CoV-2 patients were conducted using a mobile 1.5T clinical scanner (Ingenia, Philips Healthcare, Best, The Netherlands). The mobile 1.5T CMR scanner is widely clinical used and established, including the T1 mapping sequences.

**Fig. 1:**
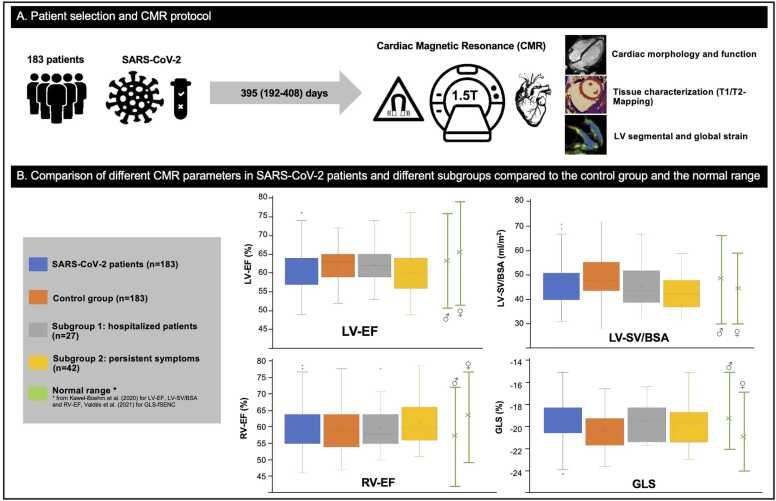
Absence of cardiac impairment in patients after SARS-CoV-2 infection: a long-term follow-up CMR study. One hundred and eighty-three patients were examined 395 (192-408) days after COVID-19 using highly efficient, contrast agent-free cardiovascular magnetic resonance imaging. Box whisker plots of common CMR parameters display the comparison of patients after SARS-CoV-2 to an age-, gender-, BMI-, and BSA-matched control group and the subgroup of previously hospitalized patients (subgroup 1) as well as to patients with persistent potentially cardiac-related symptoms (subgroup 2). The normal range for men and women is shown in green bars. Except for a few patients, functional parameters such as LV-EF, LV-SV/BSA, RV-EF, and GLS were within the normal defined range. Even in the subgroups of hospitalized patients or patients with persistent cardiac-related symptoms (shortness of breath, chest pain, palpitations, or leg edema), no clinically relevant changes were observed. *SARS-CoV-2* severe acute respiratory syndrome coronavirus 2, *CMR* cardiovascular magnetic resonance, *COVID-19* coronavirus disease 2019, *BMI* body mass index, *LV* left ventricular, *EF* ejection fraction, *SV* stroke volume, *BSA* body surface area, *RV* right ventricle, *GLS* global longitudinal strain

**Fig. 2:**
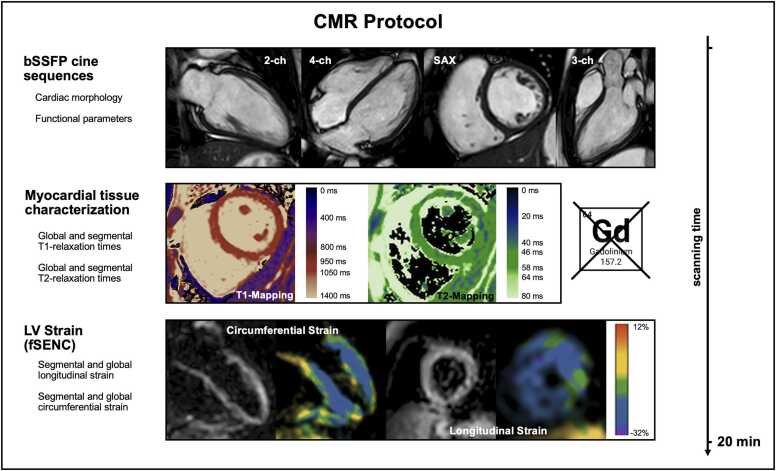
CMR protocol of patients after SARS-CoV-2 infection. Standard, highly efficient, and contrast agent-free CMR protocol of patients after SARS-CoV-2 including bSSFP cine sequences in long- and short-axis views for the assessment of morphological and functional parameters, T1- and T2-mapping sequences and LV strain (fSENC) for segmental as well as global longitudinal and circumferential strain. *CMR* cardiac magnetic resonance imaging, *SARS-CoV-2* severe acute respiratory syndrome coronavirus 2, bSSFP balanced steady-state free precession, *fSENC* Fast-Strain-ENCoded imaging, *2-ch* two chamber, *3-ch* three chamber, *4-ch* four chamber, *LV* left ventricular, *SAX* short axis

The standard examination protocol included balanced steady-state free precession cine sequences, including two-, three-, four-chamber views and a short-axis stack covering the entire ventricles. Additionally, T1 and T2 mapping with five short-axis slices of the LV and fSENC acquisitions in three short-axis (apical, midventricular, and basal) and three long-axis slices (two-, three-, and four-chamber views) were performed (Figs. 1and 2). As in previous studies described, single heartbeat acquisition was used for fSENC sequences [18]. Native myocardial T1 maps were generated from modified look-locker inversion recovery (MOLLI) images, T2 mapping was performed using a multi-echo gradient-spin-echo sequence (GraSE).

Standard CMR parameters were derived from short- and long-axis views using dedicated CMR software (cvi42, Circle Cardiovascular Imaging, Calgary, Alberta, Canada). Quantitative native T1- and T2-maps were generated by cvi42 and global T1- and T2-relaxation times were measured.

All morphological and functional parameters were analyzed in accordance with recent recommendations [19], with papillary muscles included as part of the LV volume. Pericardial effusions were evaluated and categorized according to size. Each patient was analyzed by two experienced CMR Level 3 certified investigators (>2000 CMR exams). In questionable and inconsistent cases, a third investigator was involved (>10,000 CMR exams).

### 2.5 Strain analysis

For the strain analysis of fSENC sequences, a specialized post-processing software (MyoStrain 5.2.2, Myocardial Solutions, Inc., Morrisville, North Carolina) was used. As described in previous studies, endo- and epicardial contours were drawn manually in three short axes for global longitudinal strain (GLS) and in three long axes for global circumferential strain (GCS) in end-systole [20], [21].

Segments with a longitudinal or circumferential strain of less than −17% were defined as normal, consistent with established criteria for LV strain derived from fSENC [13], [22], [23], [24]. Circumferential strain was derived from long-axis views, measuring apical cap in two-, three- and four-chamber views, apical anterior in two- and three-chamber view and apical lateral in three- and four-chamber views, resulting in 21 segments as previously described. The total number of normal segments (16 for longitudinal and 21 for circumferential strain) was divided by the total number of segments per patient (n = 37) as follows: fraction of normal myocardial segments = segments with circumferential and longitudinal strain <−17%/37.

### 2.6 Subgroup analysis

The authors defined the following subgroups. Subgroup 1 included all subjects who were hospitalized during acute infection phase. Subgroup 2 included all subjects who reported ongoing and potentially cardiac-related persistent symptoms, including shortness of breath, chest pain, palpitations, or leg edema. Subgroup 3 included all subjects who experienced persistent impaired exercise tolerance after SARS-CoV-2 infection. These individuals reported struggling with physical activities that they could previously perform. Each subgroup was separately compared against all other remaining patients in the study cohort.

### 2.7 Statistical analysis

The statistical analysis was conducted using commercially available software (MedCalc 22.016, Mariakerke, Belgium). Normal distribution was assessed using the Shapiro-Wilk test.

Descriptive statistics included mean ± standard deviation for parametric variables and median with interquartile range for nonparametric variables. To compare continuous variables between two groups, Student’s t-test and Mann Whitney U test were used if applicable. Categorical variables were compared using the Chi^2^ test.

We performed a univariable analysis to identify the most significant variables for inclusion in the multivariable model. Then, we used a stepwise forward logistic regression model to assess the incremental value of clinical and CMR parameters on group assignment. Significant variables were entered sequentially; after entering a variable in the model, it was checked and possibly removed when it became non-significant. A p-value of <0.05 was regarded as statistically significant.

## 3 Results

### 3.1 Baseline characteristics

One hundred and ninety-one patients fulfilled the inclusion criteria for the study. Of those, 8 patients were excluded due to technical issues and poor image quality (n = 6) or the abortion of the CMR scan (n = 2). The final study population consisted of 183 patients (48.1% male (88/183)) with a mean age of 48.4 ± 13.4 years. The mean scan time for the contrast agent-free CMR protocol was 24 min 49 s (±4 min 21 s). Cardiovascular risk factors were present in a minority of patients with arterial hypertension (24.0% (44/183)) and smoking (29.0% (53/183)) being the most frequent ones (Table 1). CMR scans were performed at a median of 395 (192-408) days after the diagnosis of SARS-CoV-2 infection. Compared to matched controls without SARS-CoV-2 infection, there were no significant differences in age, gender, BMI, or BSA. Significantly more SARS-CoV-2 patients were smokers compared to controls. Conversely, more controls suffered from dyslipidemia compared to the study group (Table 1).

**Table 1:** Baseline clinical characteristics as well as cardiac function and dimensions of patients after SARS-CoV-2 infection.

	SARS-CoV-2 patients (n = 183)	Controls (n = 183)	p
Baseline characteristics			
Age, year	48.4 ± 13.4	48.6 ± 13.2	0.870
Male gender, n (%)	88 (48.1)	88 (48.1)	1.000
BMI, kg/m^2^	26.2 ± 4.7	25.9 ± 3.9	0.492
BSA, m^2^	1.94 ± 0.23	1.93 ± 0.20	0.844
Heart rate at rest, bpm	65 (59-74)	70 (60-74)	0.187
Cardiovascular risk factors, n (%)			
Arterial hypertension	44 (24.0)	47 (25.7)	0.606
Current or former smoker	53 (29.0)	3 (5.8)	**<0.001**
Dyslipidemia	18 (9.8)	41 (22.4)	**<0.001**
Diabetes mellitus	5 (2.7)	6 (3.3)	0.725
Left ventricle			
LV-EF, %	60.6 ± 5.6	61.9 ± 4.3	**0.011**
LV-EDVi, mL/m^2^	76.7 ± 14.0	79.4 ± 14.6	0.076
LV-ESVi, mL/m^2^	30.5 ± 8.0	30.2 ± 8.3	0.684
LV-SVi, mL/m^2^	46.2 ± 8.2	49.5 ± 8.9	**<0.001**
LV-mass, g	91.6 ± 25.2	96.9 ± 22.5	**0.034**
Septum thickness, mm	9 (8-11)	8 (7-9)	**<0.001**
Lateral wall thickness, mm	6 (5-7)	6 (5-7)	0.966
MAPSE, mm	12 (11-13)	13 (11-14)	**<0.001**
Right ventricle			
RV-EF, %	58 (55-64)	59 (54-64)	0.947
RV-EDVi, mL/m^2^	75 (67-87)	85 (75-95)	**<0.001**
RV-ESVi, mL/m^2^	30 (25-38)	33 (28-42)	**<0.01**
RV-SVi, mL/m^2^	45 (40-51)	50 (44-56)	**<0.001**
TAPSE, mm	23.1 ± 4.0	24.3 ± 3.6	**<0.001**
LA, mm	33.4 ± 5.1	34.5 ± 6.1	0.078
RA, mm	49.5 ± 6.5	49.9 ± 5.0	0.154
Strain			
GLS (%)	−19.4 ± 1.8	−20.4 ± 1.6	**<0.001**
GCS (%)	−20.4 ± 1.5	−20.3 ± 1.5	0.526
Normal myocardial segments, n (%)*			
>80	96 (52.4)	100 (69.9)	**<0.01**
<80 and >60	73 (39.9)	43 (30.1)	0.067
<60	14 (7.7)	0 (0)	**<0.001**
Tissue characterization			
Native T1 times, (mm)	1016 ± 30	1011 ± 21†	0.317
Native T2 times, (mm)	50.9 ± 2.1	95% CI: 55.0-56.5‡	

*EDVi* indexed end-diastolic volume, *EF* ejection fraction, *ESVi* indexed end-systolic volume, *GCS* global circumferential strain, *GLS* global longitudinal strain, *LA* left atrium, *LV* left ventricular, *MAPSE* mitral annular plane systolic excursion, *RA* right atrium, *RV* right ventricle, *SVi* indexed stroke volume, *TAPSE* tricuspid annular plane systolic excursion, *SARS-CoV-2* severe acute respiratory syndrome coronavirus 2, *BMI* body mass index, *BSA* body surface area, *CI* confidence interval

Normal myocardial segments (strain) <−17%.

A p-value < 0.05 was considered as statistically significant (bold)

* Strain values available in 143 control patients

† Controls for native T1 mapping n = 11, 95% CI 995-1020 ms

‡ Reference range: 95% CI: 55.0-56.5 ms, according to Granitz et al. [17]

### 3.2 Treatment setting and clinical presentation of SARV-CoV-2 patients

The predominant acute SARS-CoV-2 symptoms were impaired exercise tolerance (78.3% (141/183)), headache (66.1% (121/183)), melalgia (65.6% (120/183)), cough (62.3% (114/183)), loss of smell or taste (59.6% (109/183)), and fever (57.9% (106/183)).

During the acute infection phase, 14.8% (27/183) of patients were hospitalized for a median duration of 8 (5-13) days. Among that group, 7.1% (13/183) of patients were admitted to intermediate care or the intensive care unit, and 7.7% (14/183) of patients required oxygen supply. Of those 14, 3 patients (1.6% (3/183)) were ventilated over a median period of 14 (12-25) days, while others received high-flow oxygen therapy (Table 2).

**Table 2:** Treatment setting and symptoms of patients during and after SARS-CoV-2 infection.

	Acute presentation in SARS-CoV-2 patients (n = 183)		
Treatment setting			
Ambulatory, n (%)	156 (85.2)		
Hospitalized for acute illness, n (%)	27 (14.8)		
Duration of hospitalization, days	8 (5-13)		
Admission to IMC/ICU, n (%)	13 (7.1)		
Duration of IMC/ICU stay, days	4 (1.4-7)		
Oxygen supply required, n (%)	14 (7.7)		
Duration of oxygen therapy, days	5.5 (3.5-9)		
Invasive ventilation required, n (%)	3 (1.6)		
Duration of invasive ventilation, days	14 (12-25)		
			
Symptoms, n (%)			Patients with persistent symptoms
Impaired exercise tolerance	141 (78.3)	→	63 (34.4)
Headache	121 (66.1)	→	2 (1.1)
Melalgia	120 (65.6)	→	20 (10.9)
Cough	114 (62.3)	→	15 (8.2)
Loss of taste or smell	109 (59.6)	→	24 (13.1)
Fever	106 (57.9)	→	2 (1.1)
Memory impairment	75 (41.2)	→	56 (30.6)
Shortness of breath	67 (36.6)	→	25 (13.7)
Chest pain	53 (29.0)	→	18 (9.8)
Palpitations	36 (19.7)	→	25 (13.7)
Leg edema	9 (4.9)	→	5 (2.7)
Syncope	4 (2.2)	→	0 (0)
Other symptoms	63 (34.8)	→	19 (10.3)

*SARS-CoV-2* severe acute respiratory syndrome coronavirus 2, *ICU* intensive care unit, *IMC* intermediate care unit

At the time of the CMR scan, the most frequently reported persistent symptoms were impaired exercise tolerance (34.4%), memory impairment (30.6%), shortness of breath (13.7%), palpitations (13.7%), loss of smell or taste (10.9%), and melalgia (10.9%). From the total study cohort, 42 patients (23.0%) reported persistent symptoms (shortness of breath, palpitations, chest pain, or leg edema) at the time of the CMR scan.

### 3.3 Cardiac function and dimensions

**Fig. 3:**
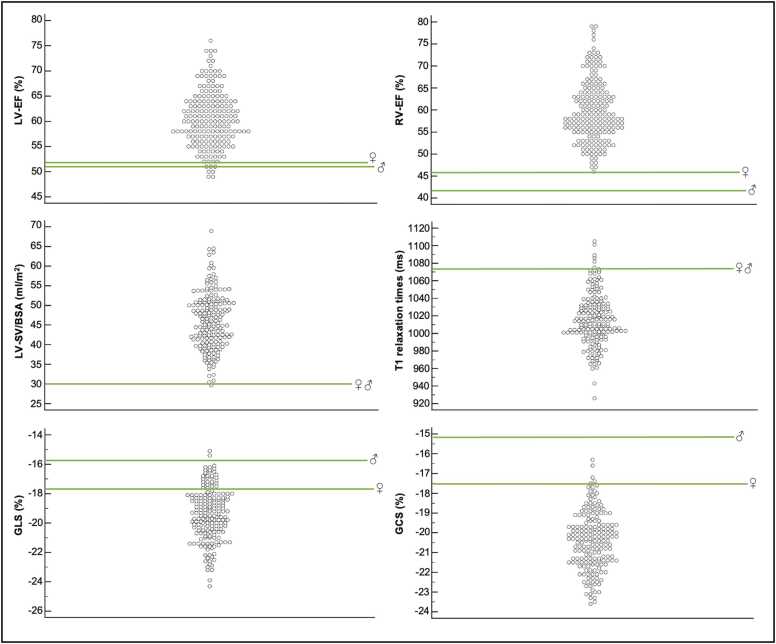
Dot plot diagrams of different CMR parameters of patients after SARS-CoV-2 infection. LV-EF, RV-EF, LV-SV/BSA, T1 relaxation times, GLS, and GCS are within the normal defined range in almost all patients after SARS-CoV-2, except for a few patients with borderline parameters. The lower or upper limit of the normal range for men and women are shown in green bars as defined by Kawel-Boehm et al. [16] and Weise Valdés et al. [25] for strain values. *CMR* cardiac magnetic resonance imaging, *SARS-CoV-2* severe acute respiratory syndrome coronavirus 2, *LV* left ventricular, *EF* ejection fraction, *SV* stroke volume, *BSA* body surface area, *RV* right ventricle, *GLS* global longitudinal strain, *GCS* global circumferential strain

Patients after SARS-CoV-2 infection showed a preserved LV ejection fraction (EF), but slightly reduced compared to controls (LV-EF (%); SARS-CoV-2: 60.6 ± 5.6; controls: 61.9 ± 4.3; p = 0.011). Indexed LV end-diastolic (LV-EDVi) and end-systolic volume (LV-ESVi) were similar in the SARS-CoV-2 group compared to controls, while indexed LV stroke volume (LV-SVi) was significantly reduced compared to controls (LV-SVi (mL/m^2^); SARS-CoV-2: 46.2 ± 8.2; controls: 49.5 ± 8.9; p < 0.001). Myocardial LV-mass was slightly reduced in SARS-CoV-2 compared to controls (LV-mass (g); SARS-CoV-2: 91.6 ± 25.2; controls: 96.9 ± 22.5; p = 0.034), while septum thickness was slightly larger in patients after SARS-CoV-2 infection compared to controls (SARS-CoV-2: 9 (8-11) mm; controls: 8 (7-9) mm; p < 0.001) (Table 1). Despite these variations, the mean LV-EF in both men and women was within normal reference range (men: lower limit (LL)-upper limit (UL): 51-76%; women: LL-UL: 52-79%) [16] (Figs. 1 and 3).

In patients post-SARS-CoV-2 infection, RV-EF was preserved and comparable to controls. RV-EDVi, RV-ESVi, and RV-SVi were significantly lower compared to controls. Left and right atrial diameters were similar across the groups (Table 1).

GLS was significantly reduced in SARS-CoV-2 patients compared to controls (SARS-CoV-2: −19.4 ± 1.8%; controls: −20.4 ± 1.6%; p < 0.001), while GCS was similar compared to controls (SARS-CoV-2: −20.4 ± 1.5%; controls: −20.3 ± 1.5%; p = 0.526).

Native T1 times (SARS-CoV-2: 1016 ± 30 ms) were similar to the values from controls from the mobile 1.5T clinical scanner (controls: 1011 ± 21 ms, 95% confidence interval (CI) 995-1020 ms, p = 0.317). Additionally, when comparing with pooled weighted reference values, the native T1 times of most patients after SARS-CoV-2 infection were within the normal range (LL-UL for 1.5T MOLLI Philips: 905-1073 ms) [26]. According to the normal reference values, seven patients had borderline or increased native T1 times. Of these, four patients showed ventricular hypertrophy, and one patient had a mitral valve prolapse with pericardial effusion. Mean T2 times (SARS-CoV-2: 50.9 ± 2.1 ms) were within the normal range compared to the reference range (95% CI: 55.0-56.5) [17]. For the T2-GraSE sequence at 1.5T using a Philips magnetic resonance imaging as used in our study, other studies found similar or even higher mean T2 values; Baeßler et al. (55.8 ± 4.2 ms) [27], Bohnen et al. (55 ± 3.1 ms) [28], Homsi et al. (54 ± 2.6 ms) [29], and Radunski et al. (57 ± 4.8 ms) [30].

Pericardial effusions were found in14.2% (26/183) patients after SARS-CoV-2 infection, with small pericardial effusions in 13.7% (25/183) and a moderate pericardial effusion in 1 patient (0.5% (1/183)).

### 3.4 Subgroup analysis

Patients who were hospitalized (14.8% (27/183)) during the acute infection phase of SARS-CoV-2 were significantly older (56.3 ± 12.2 vs 47.0 ± 13.1 years; p < 0.001) and more likely to be male (67% vs 45%; p < 0.05) compared to patients who received ambulatory treatment. Also, more patients hospitalized for SARS-CoV-2 patients suffered from diabetes mellitus (3 (11.1%) vs 2 (1.3%); p < 0.01) than SARS-CoV-2 patients who were not hospitalized. There were no relevant differences in CMR parameters regarding LV or RV function or dimensions between patients who were treated in the hospital or who were treated at home. Also, there were no relevant differences in strain, as well as native T1 or T2 times between patients who were hospitalized during the acute infection phase compared to those who were not hospitalized (Table 3).

**Table 3:** Comparison of hospitalized SARS-CoV-2 patients to patients with ambulatory treatment during acute infection.

	SARS-CoV-2 patients with prior hospitalization (n = 27)	SARS-CoV-2 patients with outpatient treatment (n = 156)	p
Baseline characteristics			
Age, years	56.3 ± 12.2	47.0 ± 13.1	**<0.001**
Male gender, n (%)	18 (67%)	70 (45%)	**<0.05**
BMI, kg/m^2^	26.4 ± 4.1	26.2 ± 4.7	0.799
Heart rate at rest, bpm	62 (55-72)	66 (60-75)	0.086
Cardiovascular risk factors, n (%)			
Arterial hypertension	8 (29.6)	36 (23.1)	0.463
Current or former smoker	6 (22.2)	47 (30.1)	0.404
Dyslipidemia	5 (18.5)	13 (8.3)	0.102
Diabetes mellitus	3 (11.1)	2 (1.3)	**<0.01**
Left ventricle			
LV-EF, %	62.3 ± 6.0	60.3 ± 5.5	0.086
LV-EDVi, mL/m^2^	73.1 ± 13.0	77.3 ± 14.1	0.155
LV-ESVi, mL/m^2^	27.9 ± 7.3	31.0 ± 8.1	0.067
LV-SVi, mL/m^2^	45.3 ± 8.1	46.3 ± 8.3	0.528
LV-mass, g	97.6 ± 20.7	90.6 ± 25.8	0.180
Septum thickness, mm	10 (9-12)	9 (8-11)	**<0.05**
Lateral wall thickness, mm	7 (5-7)	6 (5-7)	0.051
MAPSE, mm	12 (10-13)	12 (11-13)	0.525
Right ventricle			
RV-EF, %	58 (55-64)	59 (55-64)	0.942
RV-EDVi, mL/m^2^	75 (65-80)	76 (67-89)	0.259
RV-ESVi, mL/m^2^	28 (24-35)	31 (25-38)	0.402
RV-SVi, mL/m^2^	44 (38-51)	46 (41-52)	0.333
TAPSE, mm	23 (19-25)	23 (21-26)	0.951
LA, mm	35.1 ± 5.1	33.2 ± 5.0	0.066
RA, mm	51.4 ± 7.1	49.2 ± 6.4	0.113
Strain			
GLS (%)	−19.6 ± 1.6	−19.4 ± 1.8	0.632
GCS (%)	−20.2 ± 1.6	−20.4 ± 1.4	0.402
Normal myocardial segments, n (%):			
>80	15 (55.6)	81 (51.9)	0.728
<80 and >60	8 (29.6)	65 (41.7)	0.240
<60	4 (14.8)	10 (6.4)	0.130
Mapping			
Native T1 time, ms	1011 ± 37	1017 ± 29	0.368
Native T2 time, ms	51.1 ± 2.4	50.9 ± 2.0	0.641

*bpm* beats per minute, *BMI* body mass index, *EDVi* indexed end-diastolic volume, *EF* ejection fraction, *ESVi* indexed end-systolic volume, *GCS* global circumferential strain, *GLS* global longitudinal strain, *LA* left atrium, *LV* left ventricular, *MAPSE* mitral annular plane systolic excursion, *RA* right atrium, *RV* right ventricle, *SVi* indexed stroke volume, *TAPSE* tricuspid annular plane systolic excursion, *SARS-CoV-2* severe acute respiratory syndrome coronavirus 2

Normal myocardial segments (strain) <−17%.

A p-value < 0.05 was considered as statistically significant (bold)

Among patients with persistent symptoms (shortness of breath, palpitations, chest pain, or leg edema) (23.0% (42/183)), univariate logistic regression analysis revealed female gender, arterial hypertension, current or former smoking status, increased heart rate, reduced LV-mass, MAPSE, RA diameter, LV/RV-EDVi, LV/RV-ESVi, LV/RV-SVi, as well as native T1 times to be associated with persistent cardiac symptoms after SARS-CoV-2 infection (Table 4). However, in a stepwise multivariable analysis, only female gender and LV-EDVi remained independently associated with persistent symptoms (odds ratio (OR) 0.169, 95% CI 0.065-0.439, <0.001; OR 0.957, 95% CI 0.923-0.991, <0.05, respectively). In a second stepwise multivariable analysis, including cardiovascular risk factors, arterial hypertension, current or former smoking status, and LV-mass remained independently associated with persistent symptoms (OR 4.396, 95% CI 1.726-11.196, <0.05; OR 2.777, 95% CI 1.186-6.502, <0.05; OR 0.966, 95% CI 0.936-0.997, <0.05, respectively).

**Table 4:** Univariate and multivariate analysis for ongoing cardiac-related symptoms and impaired exercise tolerance after SARS-CoV-2 infection.

	Univariate analysis			Multivariate analysis models					
Ongoing cardiac symptoms	OR	95% CI	p	1. model OR	95% CI	p	2. model OR	95% CI	p
Age	1.011	0.985-1.038	0.417						
Male gender*	0.120	0.048-0.303	<0.0001	0.169	0.065-0.439	<0.001	0.309	0.081-1.170	0.084
Arterial hypertension†	2.871	1.363-6.050	<0.01				4.396	1.726-11.196	<0.05
Current or former smoker†	2.161	1.061-4.399	<0.05				2.777	1.186-6.502	<0.05
Dyslipidemia	1.792	0.629-5.106	0.275						
Diabetes mellitus	0.835	0.091-7.683	0.874						
Heart rate at rest	1.041	1.009-1.074	<0.05						
LV-EF	1.038	0.977-1.104	0.230						
LV-EDVi*	0.942	0.913-0.972	<0.001	0.957	0.923-0.991	<0.05			
LV-ESVi	0.920	0.874-0.968	<0.01						
LV-SVi	0.918	0.872-0.967	<0.01						
LV-mass*	0.957	0.939-0.976	<0.0001				0.966	0.936-0.997	<0.05
Septum thickness	0.833	0.694-0.999	<0.05						
Lateral wall thickness	0.602	0.440-0.823	<0.01						
MAPSE	0.771	0.638-0.931	<0.01						
RV-EF	1.039	0.990-1.091	0.121						
RV-EDVi*	0.946	0.919-0.974	<0.0001						
RV-ESVi	0.937	0.901-0.975	<0.01						
RV-SVi	0.908	0.861-0.958	<0.01						
RA	0.879	0.821-0.941	<0.01						
GLS	0.852	0.670-1.037	0.110						
Native T1*	1.013	1.000-1.025	<0.05						
									
Persistent impaired exercise tolerance	OR	95% CI	p	OR	95% CI	p			
Age	0.986	0.963-1.010	0.249						
Male gender*	0.451	0.236-0.861	<0.05						
Arterial hypertension	1.033	0.492-2.168	0.933						
Current or former smoker	2.010	0.964-4.191	0.063						
Dyslipidemia	0.498	0.185-1.339	0.167						
Diabetes mellitus	0.636	0.103-3.917	0.626						
LV-EF	1.010	0.954-1.069	0.740						
LV-EDVi	0.959	0.936-0.982	<0.001						
LV-ESVi	0.953	0.916-0.992	<0.05						
LV-SVi*	0.928	0.891-0.967	<0.001	0.945	0.904-0.987	<0.05			
LV-mass*	0.977	0.964-0.991	<0.0001	0.984	0.970-0.999	<0.05			
Septum thickness	0.796	0.625-1.015	0.065						
Lateral wall thickness	0.858	0.729-1.010	0.065						
MAPSE	0.911	0.782-1.060	0.226						
RV-EF	1.010	0.965-1.057	0.662						
RV-EDVi	0.970	0.951-0.990	<0.01						
RV-ESVi	0.966	0.938-0.995	<0.05						
RV-SVi*	0.933	0.896-0.973	<0.01						
RA	0.928	0.882-0.975	<0.01						
GLS	0.918	0.766-1.100	0.352						
Native T1*	1.003	0.992-1.014	0.628						

*EDVi* indexed end-diastolic volume, *EF* ejection fraction, *ESVi* indexed end-systolic volume, *GLS* global longitudinal strain, *LV* left ventricular, *MAPSE* mitral annular plane systolic excursion, *RA* right atrium, *RV* right ventricle, *SVi* indexed stroke volume, *OR* odds ratio, *CI* confidence interval, *SARS-CoV-2* severe acute respiratory syndrome coronavirus 2

* Variables used in stepwise multivariate analysis

† Variables only included in a second stepwise multivariate analysis

Overall, 69.9% (128/183) reported persistent impaired exercise tolerance after SARS-CoV-2 infection. In a univariate analysis, female gender, reduced LV-mass, RA diameter, as well as reduced indexed LV/RV-EDV, LV/RV-ESV, and LV/RV-SV were associated with persistent impaired exercise tolerance. Only indexed LV-SV and LV-mass were independently associated with persistent impaired exercise tolerance after SARS-CoV-2 infection (OR 0.945, 95% CI 0.904-0.987, <0.05; OR 0.984, 95% CI 0.970-0.999, <0.05, respectively) (Table 4).

### 3.5 Individuals with slightly reduced cardiac function

Six patients (3.3% (6/183)) (three males and three females) revealed LV-EF at the LL of the reference range (range 49-51%) (Table 5). LV-EDVi was within the normal range of the reference values from the existing literature [16]. One female patient (patient No. 5) showed elevated native T1 times (1059 ms) with borderline GLS (−16.9%).

**Table 5:** Results of cardiac function and dimensions in patients after SARS-CoV-2 infection with borderline left ventricular function.

No.	Age, years	Gender	BMI, kg/m^2^	Cardiovascular risk factors	LV-EF, %	LV-EDVi, mL/m^2^	GLS, %	GCS, %	Native T1 times, ms	Native T2 times, ms
22	53	Male	24.4	None	49	100	−19.7	−19.4	1030	53.0
32	45	Female	25.6	aHT, former smoker	50	89	−16.7	−19.4	1024	56.6
39	29	Male	25.3	Current smoker	50	104	−18.1	−19.0	981	48.4
70	54	Female	22.5	None	51	74	−17.8	−19.0	996	48.6
154	54	Female	25.8	Former smoker	51	81	−16.9	−18.2	1059	54.3
179	65	Male	28.1	None	49	90	−17.1	−21.5	1003	52.8

*aHT* arterial hypertension, *EDVi* indexed end-diastolic volume, *EF* ejection fraction, *GCS* global circumferential strain, *GLS* global longitudinal strain, *LV* left ventricular, *SARS-CoV-2* severe acute respiratory syndrome coronavirus 2, *BMI* body mass index

Normal range for left ventricular ejection fraction according to Kawel-Boehm et al. and Granitz et al. [16], [17]

## 4 Discussion

In this study, which included 183 patients after SARS-CoV-2 infection, we investigated potential cardiac functional and structural alterations in a long-term follow-up, with a specific emphasis on any functional impairment. The main findings of our study include [1] the vast majority of patients did not exhibit any pathological findings in CMR after SARS-CoV-2 infection, even after a median follow-up of 1 year. This was particularly evident when looking at cardiac function. Our study therefore suggests an absence of clinically relevant cardiac long-term alterations following SARS-CoV-2 infection. [2] CMR analyses of various subgroup analyses, particularly of SARS-CoV-2 patients after hospitalization, revealed no relevant functional impairment. This suggests that the absence of long-term changes in functional CMR parameters even applies to those who were hospitalized for SARS-CoV-2 infection. [3] We noted minor morphological deviations in patients experiencing persistent symptoms and persistent impaired exercise tolerance. These primarily involved slightly reduced LV diameters and reduced indexed LV-SV, while cardiac function remained preserved in these individuals. The clinical significance of these minor morphological deviations remains unclear but is probably negligible.

### 4.1 CMR parameters in patients after SARS-CoV-2 infection

Unlike this study, previous CMR studies reported a decline in LV systolic function, as well increased elevated myocardial native T1 and T2 values after SARS-CoV-2 infection [4], [31]. For instance, Puntmann et al. reported abnormal findings in 78 out of 100 patients [4]. But while prior studies have examined acute and post-acute myocardial changes due to SARS-CoV-2 [3], [4], [6], [32], [33], they had shorter follow-up periods (1-6 months) [31], [34] than this study. In addition, most studies examined patients with a more severe disease course and need for hospitalization [35], [36] compared to our study cohort. Myocardial involvement ranged from 26% to 60% in hospitalized patients including LV/RV dysfunction or myocarditis-like late gadolinium enhancement (LGE) patterns [3], [31], [34], [37], [38]. Non-hospitalized patients exhibited less frequent CMR abnormalities [4], [39]. By contrast, our study examined a cohort of post-SARS-CoV-2 infection patients, with the majority of whom we assessed more than 1 year after each patient`s initial infection. Moreover, our study population was more heterogeneous compared to prior studies. Many of our patients had only a mild or moderate acute infection. Our study therefore likely reflects real-world conditions more accurately, as most patients with COVID-19 did not need to be hospitalized.

Most of our study participants had normal cardiac function and structure with regards to reference values in the literature [16]. Even when comparing non-hospitalized patients to hospitalized patients, including those needing oxygen or invasive ventilation, we found no relevant differences in long-term cardiac morphology and function, as well as in LV strain and T1/T2 mapping. In subgroups with persistent cardiac symptoms, our multivariate analysis identified two factors as predictive: female gender and reduced LV-EDVi. This is consistent with prior research showing that female gender is independently associated with long COVID symptoms, including fatigue and shortness of breath [40], [41].

Reduced LV-EDVi, LV-SVi, and LV-mass were independently associated with exercise intolerance or persistent symptoms, a phenomenon not observed in previous CMR studies with patients post-SARS-CoV-2 infection [42], [43], [44]. The longer follow-up period in our study, over 1 year post-SARS-CoV-2 infection, might explain these differences. Reduced LV-SVi and LV-mass are typically found in non-athletic individuals compared to their athletic counterparts [45], [46]. This suggests that decreased physical activity post-SARS-CoV-2 infection might have contributed to these findings. Only six patients showed impaired LV-EF, while all other SARS-CoV-2 patients showed normal LV-EF. As mentioned above, LV-EF decreased only slightly, and other systolic and morphologic parameters such as LV-EDVi, GLS, GCS, and T1 and T2 values remained within the normal range. Such incidental findings are common in population-based studies and are independent of SARS-CoV-2 [47], [48], [49]. Only two patients meet the criterion of a reduced LV-EF of less than 50% in our study [50]. Since numerous studies have shown the incremental prognostic value of LV strain, the clinical relevance of this slightly reduced LV-EF appears rather low [13], [51], [52].

Pericardial effusion, previously reported in 7% to 20% of cases [4], [53], was mostly small and less prevalent in our study. In the general population, pericardial effusions are found at a rate of 6.5%, based on data derived from echocardiography [54]. In conclusion, aside from minor changes in a few individuals, most assessed morphologic and functional parameters did not reveal significant cardiac pathology in our long-term CMR follow-up.

### 4.2 Myocardial involvement of viral infections

Our subjects' infection period ran from February 2020 to March 2021. During that period, the predominant variants of SARS-CoV-2 in Germany were the wild-type and later the alpha variant [55]. Although data on virus variant distribution were limited due to limited PCR sequencing, both the wild-type and alpha variants, associated with severe disease, were prevalent in our study [56], [57]. Even in these “more aggressive” early virus variants, our results suggest that long-term cardiac complications are rare.

While certain studies have indeed reported significantly more frequent cardiac alterations in SARS-CoV-2, concomitant myocardial involvement of viral infections is not new. For example, other viral infections, such as influenza A, B, and C, have been shown to cause critical illness with myocardial involvement [58]. Autopsy findings in acute influenza patients revealed cardiac involvement, including viral myocarditis, in 10-15% of cases [59], [60]. Interestingly, recent data have shown that while the in-hospital mortality of COVID-19 myocarditis cases was reported to be higher, the likelihood of cardiogenic shock in those patients was lower than in patients with influenza myocarditis [61]. Therefore, high-risk patients should consider vaccination against serious viral infections, as demonstrated by the INVESTED trial for influenza [62]. Effective vaccination against COVID-19, similar to seasonal influenza, could protect high-risk groups from infection and associated acute cardiopulmonary events. Changes in LV systolic function, or occurrence of myocardial edema or fibrosis after COVID-19 vaccination, were not detected using CMR in asymptomatic and mildly symptomatic patients [63]. Symptoms post-COVID-19 vaccine myocarditis vary from mild to severe, including heart failure. However, numerous studies have found the incidence of post-COVID-19 vaccine myocarditis to be low, with rates varying by gender, age, and vaccine dosage [64]. Incidences of COVID-19 post-vaccination myocarditis vary between 0.08 and 15.07 per 100.000 individuals [65], [66]. Even in patients with CMR-detected post-COVID-19 vaccine myocarditis, all patients showed a normalized LV systolic function and only minimal persistent LGE after a median follow-up of 232 days [67].

Additionally, in patients with low risk for cardiac disease, a highly efficient contrast agent-free protocol could serve as a gatekeeper. In the presence of any pathological findings, the CMR protocol could be expanded to include LGE for tissue characterization. Prospective, multi-center trials are necessary to further evaluate the diagnostic and prognostic value of these highly efficient contrast agent-free CMR protocols. The use of a mobile 1.5T clinical magnetic resonance scanner allows greater flexibility and accessibility, potentially improving patient care by increasing the availability of CMR. Additionally, we efficiently scanned up to 19 patients in a single day with comparable image quality to conventional magnetic resonance scanners.

## 5 Limitations

The population of patients after SARS-CoV-2 infection was heterogeneous and relevant disease information relied on anamnestic information. Because we lacked the means of systematically excluding existing cardiac diseases, our study may have resulted in some pathological findings that were not attributable to SARS-CoV-2-related changes, but rather to a patient's prior heart disease—for instance, the few cases with borderline EF.

We also lacked information on pharmacological treatment during the acute phase of our subjects' SARS-CoV-2 infection. However, it can be assumed that hospital-admitted patients to IMC/ICU received the recommended treatment at this time (e.g. dexamethasone), corticosteroid medication (e.g. dexamethasone). Moreover, T1 values for the control group were derived from a substantially smaller group of patients, which could have increased the risk of type I error. However, after comparing native T1 times of SARS-CoV-2 patients with literature values, only seven of our subjects showed slightly increased T1 times, which were mostly associated with LV hypertrophy. Additionally, the mobile 1.5T CMR scanner is widely clinical used and established, including the T1 mapping sequences. Because no contrast agent was administered, we did not assess LGE and extracellular volume. This limitation prevented the detection of mild fibrosis and subepicardial alterations (e.g. post-COVID-19-associated myocarditis), creating a potential diagnostic gap in our study. Also, we did not evaluate native T1 times on a segmental level, which might have prevented the detection of mild myocardial tissue alterations as well. However, even if we had detected mild fibrosis, they would not have resulted in clinically relevant functional impairment in our patient cohort with a relatively long follow-up. CMR allowed to investigate functional and structural cardiac alterations of the SARS-CoV-2 patients. However, CMR does not assess arrhythmias, atherosclerotic changes, or underlying inflammatory processes due to SARS-CoV-2. Additionally, a possible inclusion bias might have caused by not including severe cases of chronic fatigue syndrome in patients after SARS-CoV-2 infections.

Although SARS-CoV-2 patients were compared with an age-, gender-, BMI-, and BSA-matched control group, there were significant differences between the two groups' smoking status and lipid profile. Smoking was found to be associated with COVID-19 disease progression in patients after SARS-CoV-2 infection [68]. Therefore, the persistent smoking status may have influenced the rate of persistent symptoms independently of SARS-CoV-2 infection.

While 12 individuals in the control group were examined in May or June 2020, after SARS-CoV-2 began occurring in Europe, none of these individuals reported symptoms of SARS-CoV-2 infection and none had traveled to high-risk regions.

Some pathologic findings found with CMR could be pre-existing and not related to SARS-CoV-2 infection.

Finally, we conducted this study in the single-center setting of a large university hospital, which may have limited the diversity of the patient population and impacted the generalizability of our findings. Collaborative multi-center studies could enhance the external validity of our results.

## 6 Conclusions

Most patients after SARS-CoV-2 infection exhibited normal cardiac functional and structural parameters. Also, previously hospital-admitted patients showed cardiac function and morphology within a normal range. Thus, the cardiac functional and structural long-term alternations of SARS-CoV-2 infections appear to be limited.

## Funding

J.S. was funded by the 10.13039/100010447German Centre for Cardiovascular Research (DZHK—Deutsches Zentrum für Herz-Kreislauf-Forschung).

## Author contributions

Henning Steen: Writing—review and editing, Supervision, Methodology, Funding acquisition, Formal analysis, Data curation, Conceptualization. Lukas Weberling: Writing—review and editing, Investigation, Formal analysis, Data curation. Norbert Frey: Writing—review and editing, Writing—original draft, Visualization, Validation, Supervision, Software, Resources, Project administration, Methodology, Investigation, Funding acquisition, Formal analysis, Data curation, Conceptualization. Andreas Ochs: Writing—review and editing, Writing—original draft, Visualization, Validation, Supervision, Software, Methodology, Investigation, Formal analysis, Data curation, Conceptualization. Florian André: Writing—review and editing, Writing—original draft, Visualization, Validation, Supervision, Software, Resources, Project administration, Methodology, Investigation, Funding acquisition, Formal analysis, Data curation, Conceptualization. Marc Zahlten: Writing—review and editing, Investigation. Jannick Heins: Writing—review and editing, Investigation, Data curation. Christian Stehning: Writing—review and editing, Supervision. James G. Whayne: Investigation. Claudia M. Denkinger: Writing—review and editing, Conceptualization. Evangelos Giannitsis: Writing—review and editing, Supervision, Resources, Project administration, Funding acquisition, Conceptualization. Sebastian J. Buss: Writing—review and editing, Supervision. Uta Merle: Resources, Investigation, Conceptualization. Janek Salatzki: Writing—review and editing, Writing—original draft, Visualization, Validation, Supervision, Software, Methodology, Investigation, Formal analysis, Data curation, Conceptualization.

## Declaration of competing interests

The authors declare the following financial interests/personal relationships which may be considered as potential competing interests: James G. Whayne reports a relationship with Myocardial Solutions Inc., Morrisville, North Carolina, United States that includes employment. Christian Stehning reports a relationship with Philips Clinical Science, Hamburg, Germany that includes employment. The other authors declare that they have no known competing financial interests or personal relationships that could have appeared to influence the work reported in this paper.
